# Macrophage depletion alters bacterial gut microbiota partly through fungal overgrowth in feces that worsens cecal ligation and puncture sepsis mice

**DOI:** 10.1038/s41598-022-13098-0

**Published:** 2022-06-04

**Authors:** Pratsanee Hiengrach, Wimonrat Panpetch, Ariya Chindamporn, Asada Leelahavanichkul

**Affiliations:** 1 Department of Microbiology, Faculty of Medicine, Chulalongkorn University, Bangkok, 10330 Thailand; 2 Translational Research in Inflammation and Immunology Research Unit (TRIRU), Department of Microbiology, Chulalongkorn University, Bangkok, 10330 Thailand; 3 Antimicrobial Resistance and Stewardship Research Unit, Department of Microbiology, Chulalongkorn University, Bangkok, 10330 Thailand; 4 Nephrology Unit, Department of Medicine, Faculty of Medicine, Chulalongkorn University, Bangkok, 10330 Thailand; 5 Immunology Unit, Department of Microbiology, Chulalongkorn University, Bangkok, 10330 Thailand; 6 Mycology Unit, Department of Microbiology, Chulalongkorn University, Bangkok, 10330 Thailand

**Keywords:** Cytokines, Infection, Inflammation, Bacteria, Fungi, Immunology, Microbiology, Medical research

## Abstract

Because macrophage dysfunction from some emerging therapies might worsen gut-derived sepsis, cecal ligation and puncture (CLP) sepsis are performed in mice with clodronate-induced macrophage depletion. Macrophage depletion (non-sepsis) increased fecal Ascormycota, with a subtle change in bacterial microbiota, that possibly induced gut-barrier defect as *Candida pintolopesii* and *Enterococcus faecalis* were identified from blood. Sepsis in macrophage-depleted mice was more severe than sepsis control as indicated by mortality, cytokines, organ injury (liver, kidney, and spleen), gut-leakage (FITC-dextran), fecal Proteobacteria, and blood organisms (bacteria and fungi). Lysate of *C. pintolopesii* or purified (1 → 3)-β-d-glucan (BG; a major component of fungal cell wall) enhanced growth of *Klebsiella pneumoniae* and *Escherichia coli* that were isolated from the blood of macrophage-depleted CLP mice implying a direct enhancer to some bacterial species. Moreover, the synergy of LPS and BG on enterocytes (Caco-2) (Transepithelial electrical resistance) and neutrophils (cytokines) also supported an influence of gut fungi in worsening sepsis. In conclusion, macrophage depletion enhanced sepsis through the selectively facilitated growth of some bacteria (dysbiosis) from increased fecal fungi that worsened gut-leakage leading to the profound systemic responses against gut-translocated LPS and BG. Our data indicated a possible adverse effect of macrophage-depleted therapies on enhanced sepsis severity through spontaneous elevation of fecal fungi.

## Introduction

Sepsis, the dysfunction of immune responses against systemic infection, is a serious healthcare problem worldwide with significant morbidity and mortality^1,2^. The gut microbiota refers to trillions of microbes in the GI tract, including bacteria, fungi, viruses, and archaea, that live in a symbiotic relationship with the host and are regulated by the host's immunity^3^. In immune-compromised hosts^4^, intestinal dysbiosis (microbial community imbalance) and gut permeability defect (gut leakage) could all lead to systemic infection via gut bacterial translocation^5^. Among all gut organisms, fungi, especially *Candida albicans* in human intestines, are the second most abundant organisms (the highest gut organisms are Gram-negative bacteria) that could be facilitated in several conditions, including antibiotic administration and chronic intestinal inflammation^5^. Although studies on the influence of gut fungi or (1 → 3)-β-d-glucan (BG), a major molecule of the fungal cell wall, on bacterial sepsis is still less, *C. albicans* administration in mice worsens sepsis models through profound inflammation from LPS-BG synergy^6,7^. However, *Candida* non-albicans are mentioned as the common fungi in mouse intestines^8^ and studies on the models with self-emerging gut fungi might provide clearer evidence on gut fungal impact.

On the other hand, macrophages are the sentinel immune cells in nearly all organs that, at least in part, critically control the enteric microbes^9^ and macrophage abnormalities weaken the host's capacity to resist fungal infections^10,11^. Macrophage is one of the major immune cells for the control of fungi, especially the yeast-formed fungi, as several immune evasion mechanisms against macrophages are evolutionarily developed by several fungi^10,12^. Indeed, macrophage phagocytosis is a well-known important fungicidal process^13,14^, especially against *C. albicans*, an important fungal gut microbiota in human^15^. As the largest pool of macrophages in the body, intestinal macrophages play a critical role in maintaining intestinal homeostasis by regulating the host-microbiota symbiosis^16^. Additionally, intestinal macrophages control the abundance of bacteria and fungi, limit the absorption of microbial molecules and maintain gut integrity^17,18^. Although the enhanced absorption of fungal products that induce enterocyte cell death and gut barrier damage after macrophage depletion has been demonstrated^18^, an impact of the depletion on gut micro-organisms has never been tested. Moreover, several emerging clinical interventions against inflammatory diseases and cancers interfere with macrophage functions^19^ that might affect sepsis and gut microbiota (bacteria and fungi) through the dominant facilitation on some gut organisms than others. Despite the well-known importance of macrophage in sepsis^20^, studies on sepsis and gut microbiota in the conditions with macrophage dysfunctions or depletion are still less^21,22^. The information on infection and gut microbiota during macrophage depletions might be useful for the upcoming interventions on macrophage functions. Hence, macrophage depletion possibly alters fungi and bacteria in the gut that might worsen sepsis severity. Accordingly, liposomal clodronate promotes macrophage death by inhibiting the mitochondrial ADP/ATP translocase enzyme and is often employed in macrophage depletion mouse models, which effectively depletes 80–90 percent of macrophages after just one intravenous or intraperitoneal dose^23^.

Here, we hypothesized that macrophage depletion causes an ineffective control in balancing gut microbiota, especially gut fungi, that might enhance sepsis severity. As such, cecal ligation and puncture (CLP) surgery was performed on macrophage-depleted mice using fecal microbiome analysis and other several investigations.

## Materials and methods

### Animal and ethics

Male 8-week-old C57BL/6 mice, weighing 20–25 g, were purchased from Nomura Siam International, Pathumwan, Bangkok, Thailand. All mice were randomly divided into 10 mice per group (5 mice per cage). The mice were housed under in a controlled-temperature environment (24 ± 2 °C), with a 12-h light–dark cycle (light from 7:00 a.m. to 7:00 p.m.). All mice received food and water ad libitum. Animal procedures were performed in adherence to US National Institutes of Health guidelines and followed the 8th Edition of the Guide for Care and Use of Experimental Animals, published by the National Research Council of the National Academies (2011; available at https://grants.nih.gov/grants/olaw/guide-for-the-care-and-use-of-laboratory-animals.pdf), as well as the Animal Research: Reporting of In Vivo Experiments (ARRIVE) guidelines. All the procedures were reviewed and approved by the Institutional Animal Care and Use Committee of the Faculty of Medicine, Chulalongkorn University, Bangkok, Thailand (animal protocol number SST018/2563).

### Sepsis model with macrophage depletion

Macrophage depletion with liposomal clodronate injection was used to assess the significance of macrophages in sepsis following a previously described methodology^24^. Briefly, liposomal clodronate (Clod) (5 mg/mL) (Encapsula Nanoscience, Nashville, TN, USA) at 200 μL/ 25 g mouse or control liposomes were injected at day 0, 2, 4, and 6 before sepsis induction to induce sustained monocyte depletion. At 24 h after the last dose of Clod, the sepsis model was performed by cecal ligation and puncture (CLP) surgery as previously described^25,26^. In brief, the cecum was ligated at 12 mm from cecal tip, punctured twice with a 21-gauge needle, and gently pushed to express a little amount of fecal material before being placed back into the abdominal cavity. In sham mice, the cecum was only identified through abdominal incision before suturing layer by layer with 6-0 nylon sutures. Mice were euthanized at 24 h post-CLP by cardiac puncture under isoflurane anesthesia with sample collection (organs, blood, and feces from descending colon). Serum was kept at − 60 °C and organs were fixed in 10% formalin before further analyses. Other groups of mice were observed for survival analysis.

### Mouse sample analysis and gut permeability measurement

For peripheral blood leukocytes, blood was mixed with 3% v/v of acetic acid for red blood cell lysis in a ratio of blood and acetic acid at 1:20 by volume before counting with a hemocytometer^27^. The percentage of neutrophils, monocytes, and lymphocytes was determined on Wright-stained blood smear slides. Hematocrit (Hct) was measured by the microhematocrit method with the Coulter Counter (Hitachi 917; Boehringer Mannheim, Indianapolis, IN, USA)^28^. Renal injury was determined by blood urea nitrogen and serum creatinine using QuantiChrom Urea-assay (DIUR-500) and Creatinine-Assay (DICT-500), respectively. Meanwhile, liver injury was evaluated by EnzyChrom ALT assay (EALT-100, BioAssay, Hayward, CA, USA). Serum cytokines were determined by enzyme-linked immunosorbent assays (ELISA) (Invitrogen, Carlsbad, CA, USA). Serum lipopolysaccharide (LPS), an indicator of sepsis severity, was measured by HEK-Blue LPS Detection (InvivoGen, San Diego, CA, USA). Gut permeability was determined by fluorescein isothiocyanate dextran (FITC-dextran) assay and serum (1 → 3)-β-d-glucan (BG) following previous publications^25,29,30^. The spontaneous detection of pathogen molecules (LPS and BG; the molecular components of gut organisms), fungemia and bacteremia without systemic infection (sham surgery), and the presence of non-intestinal absorbable molecules in serum after an oral administration indicate gut permeability defect (gut leakage)^25,29,30^. As such, FITC-dextran, an intestinal nonabsorbable molecule with molecular weight (MW) 4.4 kDa (Sigma-Aldrich, St. Louis, MO, USA) at 12.5 mg per 25 g mouse was orally administered at 3 h before serum FITC-dextran detection by a Fluorospectrometer (NanoDrop 3300) (ThermoFisher Scientific, Wilmington, DE, USA). Serum BG was measured and Fungitell (Associates of Cape Cod, Inc., East Falmouth, MA, USA), respectively. The serum BG was reported as 0 when the values of BG were lower than 7.8 pg/mL because they were below the standard curve's lower limit. For the organism burdens, 25 mL of blood was directly spread onto blood agar or 0.1% chloramphenicol in Sabouraud dextrose agar (SDA) (Oxoid, Hampshire, UK) for the determination of bacteria and fungi, respectively, and incubated at 37 °C for 24 h and 35 °C for 72 h, respectively, before colony enumeration. The colonies were identified by mass spectrometry analysis (Vitek MS; bioMérieux SA, Marcy-l’Etoile, France) according to the routine hospital protocols. In addition, fecal samples were suspended with PBS at a ratio of 100 mg per 1 mL, serially diluted before plating onto SDA (Oxoid), and incubated at 35 °C for 72 h before colony enumeration for fungi.

### Histological analysis and immunohistochemistry imaging

The semi-quantitative evaluation of renal and gastrointestinal histology on paraffin-embedded slides was performed after 10% neutral buffered formalin fixation, followed by Hematoxylin and Eosin (H&E) staining at 200 × magnification in 10 randomly selected fields for each mouse as previously described^31^. Briefly, the renal injury was defined as tubular epithelial swelling, loss of brush border, vacuolar degeneration, necrotic tubules, cast formation, and desquamation using the following scoring method: 0, area of damage < 5%; 1, area of damage 5–10%; 2, area of damage 10–25%; 3, area of damage 25–50%; and 4, area of damage > 50%. Intestinal injury score on H&E stained slides at 200 × magnification was determined^27^ based on mononuclear cell infiltration, epithelial hyperplasia, goblet cell reduction, and epithelial vacuolization using the following scores; 0: leukocyte < 5% and no epithelial hyperplasia (< 10% of control); 1: leukocyte infiltration 5–10% or hyperplasia 10–25%; 2: leukocyte infiltration 10–25% or hyperplasia 25–50% or reduced goblet cells (> 25% of control); 3: leukocyte infiltration 25–50% or hyperplasia > 50% or intestinal vacuolization; 4: leukocyte infiltration > 50% or ulceration. Additionally, the effectiveness of macrophage depletion was determined by immunohistochemistry staining on 4 µm thick paraffin sections using an antibody against F4/80 molecule, a unique marker of murine macrophages (Invitrogen, Waltham, Massachusetts, USA)^24^. In parallel, an anti-active caspase-3 antibody (Cell Signaling Technology, Beverly, MA, USA) was used for immunohistochemistry, which were then evaluated in 10 randomly selected 200 × magnified fields as previously described^30^. The spleen apoptosis score was expressed as positive cells per high-power field.

### Fecal microbiome analysis

Feces from each mouse (0.25 g per mouse) from different cages in each experimental group were collected for the microbiota analysis following a previous protocol^32^. In short, metagenomic DNA was extracted by DNeasy PowerSoil Kit (Qiagen, Maryland, USA) using the Universal prokaryotic 515F (forward; (5′-GTGCCAGCMGCCGCGGTAA-3′) and 806R (reverse; 5′-GGACTACHVGGGTWTCTAAT-3′), with the Illumina adapter and Golay barcode sequences for 16S rRNA gene V4 library construction in Miseq300 platform (Illumina, San Diego, CA, USA). The raw sequences and operational taxonomic unit (OTU) were classified following Mothur’s standard operating platform^33^. In parallel, to identify the taxonomic profiles of fungal microbiota (mycobiome) in feces, the DNA was extracted from fecal samples. The universal eukaryotic primers; ITS3 (forward; 5′-GCATCGATGAAGAACGCAGC-3′) and ITS4 (reverse; 5′-TCCTCCGCTTATTGATATGC-3′) were used for identifying the gut mycobiota. The fungal DNA library was sequenced using the Miseq system (Illumina, San Diego, CA, USA) at Omics Sciences and Bioinformatics Center, Chulalongkorn University. Forward and reverse primers were removed from raw sequences using cutadapt v 1.18. and trimmomatic v 0.39 with the sliding window option to trim individual sequences where the average quality scores less than 15 across 4 base pairs. To identify the composition of fungi in fecal samples, amplicon sequence variants (ASVs) were analyzed using the QIIME2 plugin DADA2 pipeline. Unclassified phylum or higher fungal classification was removed. To confirm non-fungal origin, the fungal classification was analyzed by a BLAST-based tool (https://blast.ncbi.nlm.nih.gov) (National Center for Biotechnology Information, Bethesda, MD, USA). The 16S rRNA and ITS3/4 sequences in this study were deposited in an NCBI open access Sequence Read Archive database with accession number PRJNA765503 (https://www.ncbi.nlm.nih.gov/sra/?term=PRJNA765503).

### Gene expression of bacteria in feces

To explore the correlation between gut bacteria and CLP, the total DNAs were extracted by a QIAamp fast DNA Stool Mini Kit (Qiagen, Hiden, Germany) with different primers for the 16S rRNA gene for real-time PCR (QuantStudio™ Design & Analysis Software) following a previous publication^34^. The primers are as followed; total bacteria (16s rRNA of bacteria V3–V4 region) (forward; 5′-CCTACGGGNGGCWGCAG-3′, reverse; 5′-GACTACHVGGGTATCTAATCC-3′), total Gram-negative bacteria (16s rRNA of Gram negative bacteria) (forward; 5′-GGAGGAAGGTGGGGATGACG-3′, reverse; 5′-ATGGTGTGACGGGCGGTGTG-3′), *Klebsiella* spp. (KP16) (forward; 5′-GCAAGTCGAGCGGTAGCACAG-3′, reverse; 5′-CAGTGTGGCTGGTCATCCTCTC-3′), *Salmonella* spp. (STM4497) (forward; 5′-AACAACGGCTCCGGTAATGAGATTG-3′, reverse; 5′-ATGACAAACTCTTGATTCTGAAGATCG-3′), *Bacteroides* spp. (Bac) (forward; 5′-GGCGCACGGGTGAGTAAC-3′, reverse; 5′-TGTGGGGGACCTTCCTCTC-3′), *Lactobacillus* spp. (L341bp) (forward; 5′-AGCAGTAGGGAATCTTCCA-3′, reverse; 5′-CACCGCTACACATGGAG-3′), and *Akkermansia* spp. (AM) (forward;5′-CAGCACGTGAAGGTGGGGAC-3′, reverse; 5′-CCTTGCGGTTGGCTTCAGAT-3′). Using the cycle threshold (Ct) at 40 as a reference negative value, the results of bacterial abundance in feces were demonstrated by the difference from the negative Ct by with the following equation; the Ct value of bacterial abundance = 40 − the measured Ct value.

### Bacterial abundance in feces and isolated bacterial experiments

Because the presence of intestinal fungi might be associated with an alteration of bacterial abundance in gut^6^, the ex vivo experiments (incubation of feces outside of the mice) with the fungi using *C. albicans* (90028) (ATCC, Manassas, VA, USA) or *C. pintolopesii* (isolated from the blood of Clodronate-administered mice) were performed. As such, feces from normal 8 weeks old mice were collected by placing the mice in a metabolic cage (Hatteras instrument, Cary, NC, USA). Then, feces (0.01 g) or the isolated bacteria from the blood of Clodronate-administered mice (*E. faecalis*, *K. pneumoniae* and *E. coli*) at 1 × 10^9^ CFU were mixed by PBS alone or in the lysate of heat-killed *C. albicans* or *C. pintolopesii* for 24 h in 37 °C before plating onto blood agar (Oxoid) in several dilutions for 24 h at 37 °C prior to the colony enumeration. For fungal lysate preparation, the fungi were cultured in SDA (Oxoid) before counting with 0.4% Trypan blue in a hemocytometer. After that, the heat-killed fungi (1 × 10^6^ CFU) were prepared by immersion in a water bath at 60 °C for 1 h, sonicated (pulse on and off at 20 and 5 s, respectively) for 1 h on ice (Sonics Vibra Cell, VCX 750, Sonics & Materials Inc., Newtown, CT, USA) until a homogenous solution was formed. The sample supernatant after centrifugation was used as the fungal lysate. In parallel, a purified BG (Pachyman, Megazyme, Bray, Ireland) in different doses or PBS control were used as a representative fungal molecule in the lysate and were mixed with the samples (feces or the isolated bacteria) as formerly described. Notably, BG is a major component of the fungal cell wall and in the fungal lysate^6^. Among several possibilities, the digestion properties of fungi (or BG) of some bacteria might be responsible for the higher abundance of such bacteria with the presence of gut fungi.

### Supernatant cytokines of enterocytes and neutrophils

An impact of the major components of bacteria and fungi upon enterocytes and neutrophils, another important innate immune cell, was explored. As such, human colorectal adenocarcinoma cells, Caco-2 (ATCC HTB-37, USA) were maintained in supplemented Dulbecco’s modified Eagle medium (DMEM) at 37 °C under 5% CO2 and sub-cultured before use in the experiments. Lipopolysaccharide (LPS), a major component of Gram-negative bacteria in the gut, from *E. coli* O26:B6 (Sigma-Aldrich) at 100 ng/mL with or without BG (Pachyman) (Megazyme) at 100 µg/mL were incubated with the enterocytes for 24 h before determination of supernatant IL-8 using ELISA (Quantikine immunoassay; R&D Systems, Minneapolis, MN, USA).

In parallel, neutrophils were isolated from the healthy volunteers with informed consent as previously described^35^. All blood samples were collected from healthy donors under an approved protocol by the Ethical Institutional Review Board, Faculty of Medicine, Chulalongkorn University, according to the Declaration of Helsinki, with written informed consent (IRB No. 426/63, COA No. 738/2020). In brief, neutrophils were isolated from heparinized blood using PolymorphPrep (Progen, Grimsby, England, UK), according to the manufacturer’s instructions and red blood cells were removed by hypotonic lysis buffer. Neutrophils were resuspended in RPMI 1640 media. Then, LPS from *E. coli* O26:B6 (Sigma-Aldrich) at 100 ng/mL with or without BG (Pachyman) (Megazyme) at 100 µg/mL were incubated with neutrophils (2.5 × 10^5^ cells/mL) for 4 h before determination of supernatant cytokines (TNF-α, IL-6, and IL-10) using ELISA (Quantikine immunoassay) (R&D Systems).

### Gene expression of enterocytes

To explore the impact of LPS and BG on enterocyte responses, the RNA from stimulated Caco-2 cells was prepared using FavorPrep Tissue total RNA purification Mini Kit (Favorgen Biotech Corp, Vienna, Austria) and cDNA was synthesized by cDNA Synthesis assay (Thermo Fisher Scientific, Wilmington, DE, USA) before the detection by SYBR green-based real-time polymerase chain reaction (PCR) (Thermo Fisher Scientific, Wilmington, DE, USA). The oligonucleotide primers for the experiment were (i) Toll-like receptor 2 (*TLR-2*); forward 3′-TCCTCCAATCAGGCTTCTCTGTCTT-5′ and reverse 3′-CTCGCAGTTCCAAACATTCC-5′, (ii) Toll-like receptor 4 (*TLR-4*); forward 3′-CACAGACTTGCGGGTTCTAC-5′ and reverse 3′-AGGACCGACACACCAATGATG-5′, (iii) *Dectin-1*; forward 5′-GAACCACAGTCAACCCACAC-3′ and reverse 5′-CCAGTTGCCAGCATTGTCTT-3′, and (iv) Glyceraldehyde 3-phosphate dehydrogenase (*GAPDH*; a house-keeping gene); forward 5′-GTGAAGGTCGGTGTCAACGGATTT-3′ and reverse 5′-CACAGTCTTCTGAGTGGCAGTGAT-3′ following the previous protocol^36^. The results were demonstrated in terms of relative quantitation of the comparative threshold (delta-delta Ct) method (2 − ∆∆Ct) as normalized by *GAPDH*.

### Transepithelial electrical resistance (TEER)

The integrity of monolayer enterocytes in different conditions was determined by TEER using Caco-2 cells (HTB-37) (ATCC) at 5 × 10^4^ cells per on the upper compartment of 24-well Boyden chamber trans-well plate in DMEM-high glucose supplemented with 20% fetal bovine serum (FBS), 1% HEPES, 1% sodium pyruvate and 1.3% Penicillin/Streptomycin for 15 days to establish the confluent monolayer. Then, LPS from *E. coli* O26:B6 (Sigma-Aldrich) at 100 ng/mL with or without BG (Pachyman) (Megazyme) at 100 µg/mL were incubated for 4 h before TEER determination using an epithelial volt-ohm meter (EVOM-2, World precision instruments, Florida, USA) by placing the electrodes in the supernatant at the basolateral and apical chamber. The TEER value in media culture without cells was used as a blank and was subtracted from all measurements. The unit of TEER was ohm (Ω) × cm^2^.

### Statistical analysis

Mean ± standard error (SE) was used for data presentation. The differences between groups were examined for statistical significance by one-way analysis of variance (ANOVA) followed by Tukey’s analysis or Student’s *t-*test for comparisons of multiple groups or 2 groups, respectively. For comparison of the time-point data, the repeated measure ANOVA was used. All statistical analyses were performed with SPSS 11.5 software (SPSS, IL, USA) and Graph Pad Prism version 7.0 software (La Jolla, CA, USA). A *p*-value of < 0.05 was considered statistically significant.

## Results

### Increased fecal fungi with a subtle change on bacterial microbiota in feces of mice with macrophage depletion

**Figure 1:**
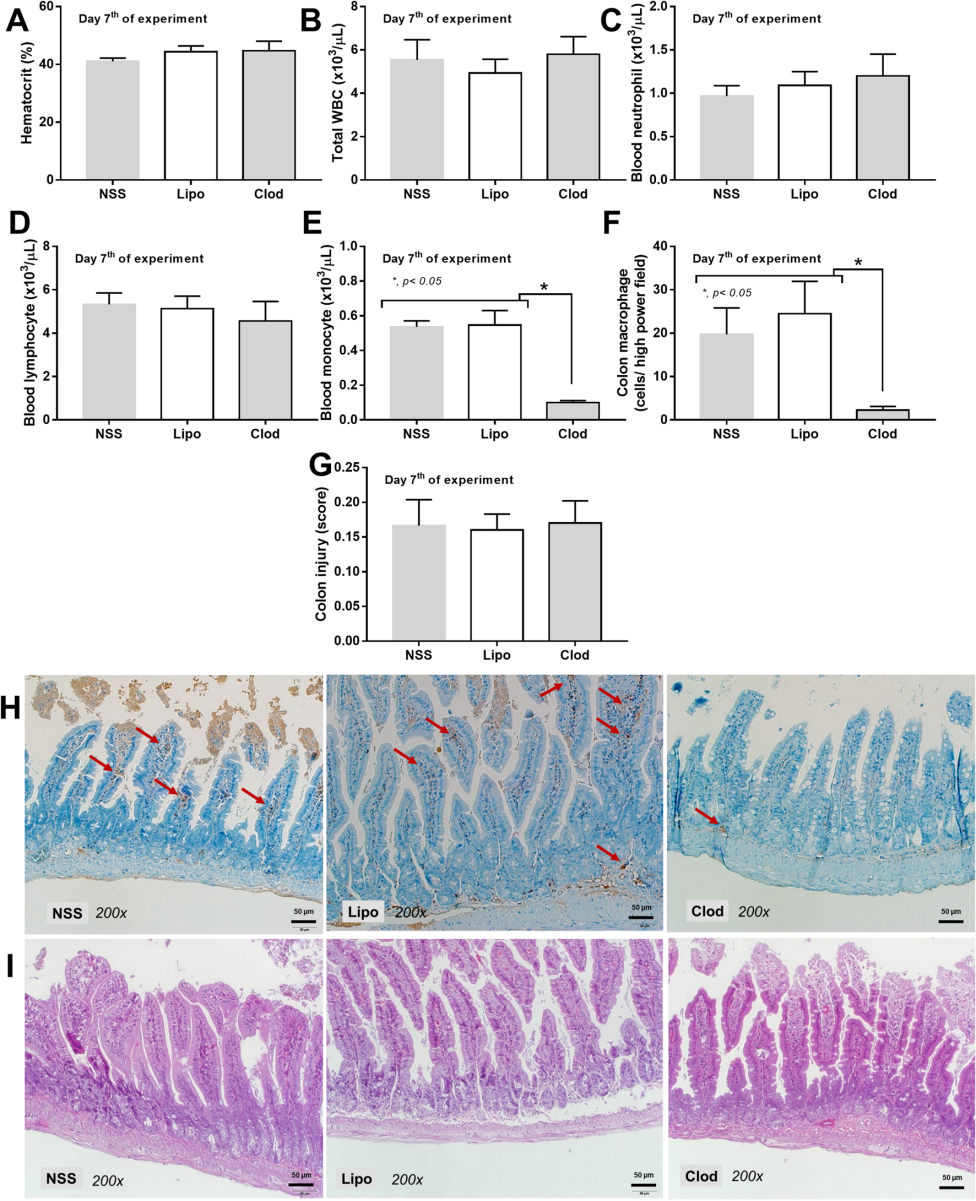
Characteristics of mice with the intraperitoneal administration by normal saline (NSS), liposome control (Lipo), or liposomal clodronate (Clod) as indicated by hematocrit and peripheral blood leukocytes, including total white blood cells (WBC), neutrophils, lymphocytes, and monocytes (**A**–**E**), macrophages in the colon (positive F4/80 cells from immunohistochemistry analysis) (**F**), colon injury score (**G**), and representative pictures of colons with immunohistochemistry and Hematoxylin and Eosin (**H**, **E**) stained slides (**H**, **I**) are demonstrated (n = 5–7/group). Arrows indicate F4/80 positive cells in the colon.

**Figure 2:**
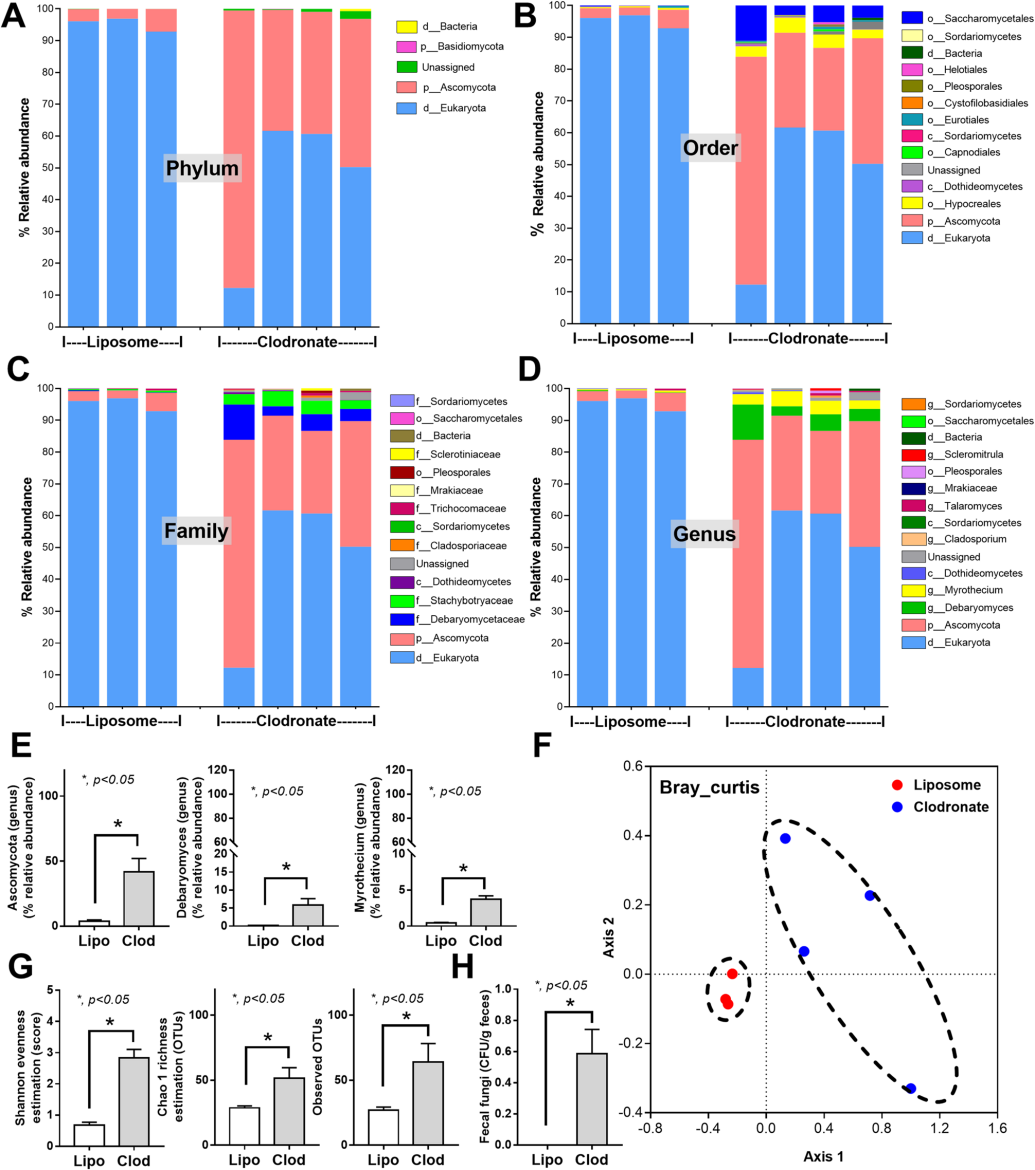
Fecal fungal microbiome (mycobiome) analysis from feces of mice with liposomal clodronate (Clod) or liposome control (Lipo) as indicated by the relative abundance in phylum, order, family and genus (**A**–**D**), graph demonstration of abundance of some genus (**E**), beta diversity (Bray Curtis) (**F**), alpha diversity (Shannon, Chao and observed total OTUs) (**G**) are demonstrated. Additionally, fecal fungal abundance by culture method (**H**) are also indicated.

**Figure 3:**
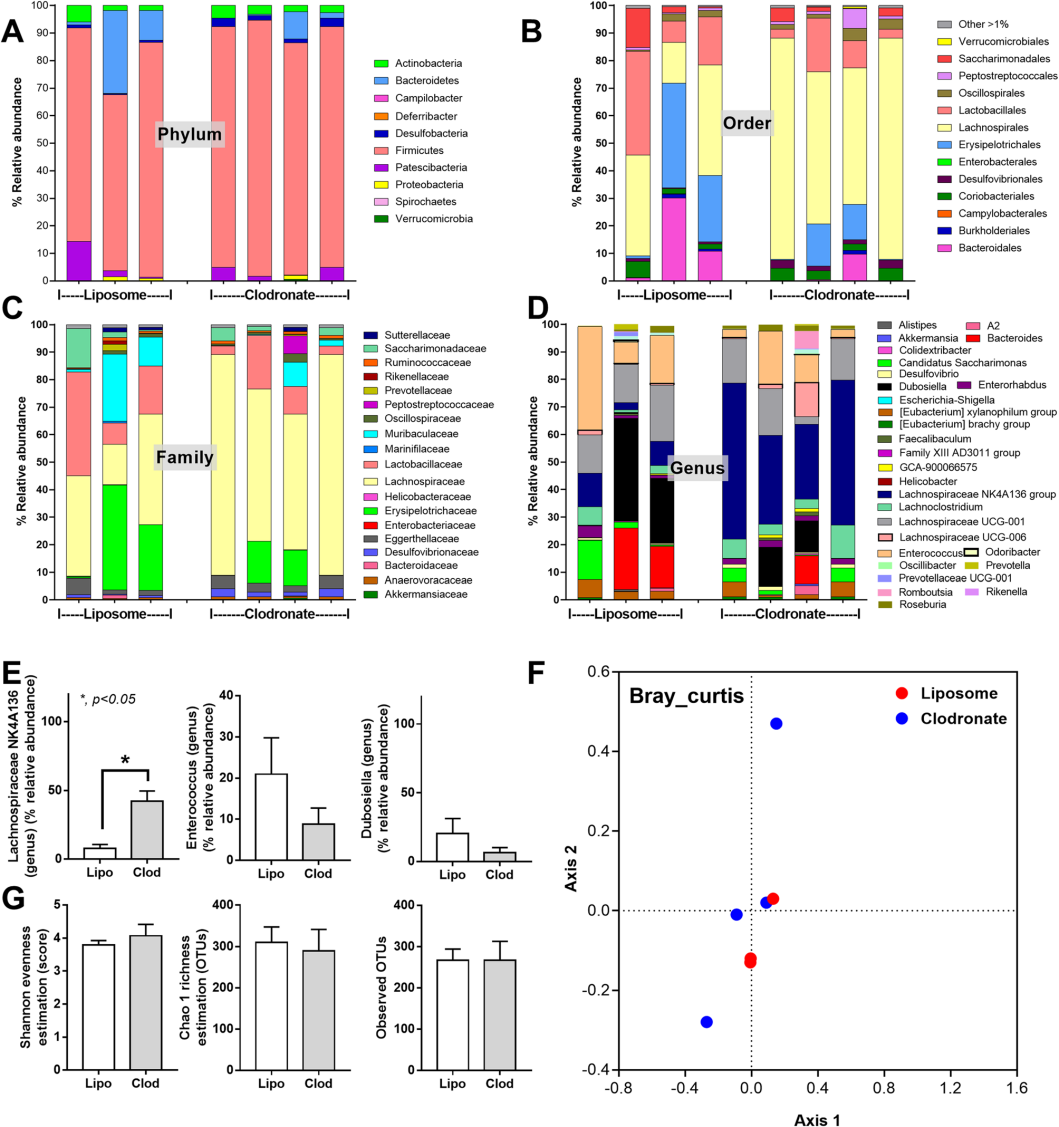
Fecal bacterial microbiome analysis from feces of mice with liposomal clodronate (Clod) or liposome control (Lipo) as indicated by the relative abundance in phylum, order, family and genus (**A**–**D**), graph demonstration of abundance of some genus (**E**), beta diversity (Bray Curtis) (**F**), alpha diversity (Shannon, Chao and observed total OTUs) (**G**) are demonstrated.

Macrophage depletion by liposomal clodronate administration was demonstrated by a decrease in peripheral blood monocytes, but not other leukocytes nor erythrocytes, and F4/80 positive cells in mouse colons without colonic mucosal injury (Fig. 1A–I). Due to the importance of macrophages in maintaining the intestinal host-microbiota symbiosis^16^, fecal microbiota analysis on fungi and bacteria was performed in macrophage-depleted mouse feces. With macrophage depletion (non-sepsis), fecal fungi in Ascomycota, Debaryomyces, and Myrothecium together with the beta diversity (Bray–Curtis) (Fig. 2A–F), alpha diversity (Shannon and Chao 1 analysis), and observed total operational taxonomic units (OTUs; cluster of similar sequence variants of the marker gene sequence) (Fig. 2G) were predominantly increased when compared with the liposome control group (Fig. 2A–G). Additionally, fecal fungi could be detected by culture only in feces of macrophage-depleted mice (Fig. 2H). These data supported the increased fecal fungal abundance (observed OTUs and fecal culture) (Fig. 2G, H), especially Ascomycota group (a group that including *Candida* spp.), in macrophage-depleted mice that was obviously different from control as the separated 2 groups of data in beta diversity analysis (Fig. 2F). On the other hand, an only alteration in the fecal bacterial microbiome of macrophage-depleted mice compared with control was an increase in Firmicutes bacteria (a dominant bacterial group in regular mice with potential benefits^37^), in particular Lachnospiraceae, without the difference in neither the diversity (alpha and beta) nor the observed OTUs (Fig. 3A–G). Notably, the beta diversity analysis of fecal bacterial microbiome between macrophage-depleted versus control mice could not be easily separated into 2 groups (Fig. 3F) implying a similarity in fecal bacterial analysis between groups.

### More severe cecal ligation and puncture (CLP) sepsis in mice with macrophage depletion, an impact of gut permeability defect and dysbiosis

**Figure 4:**
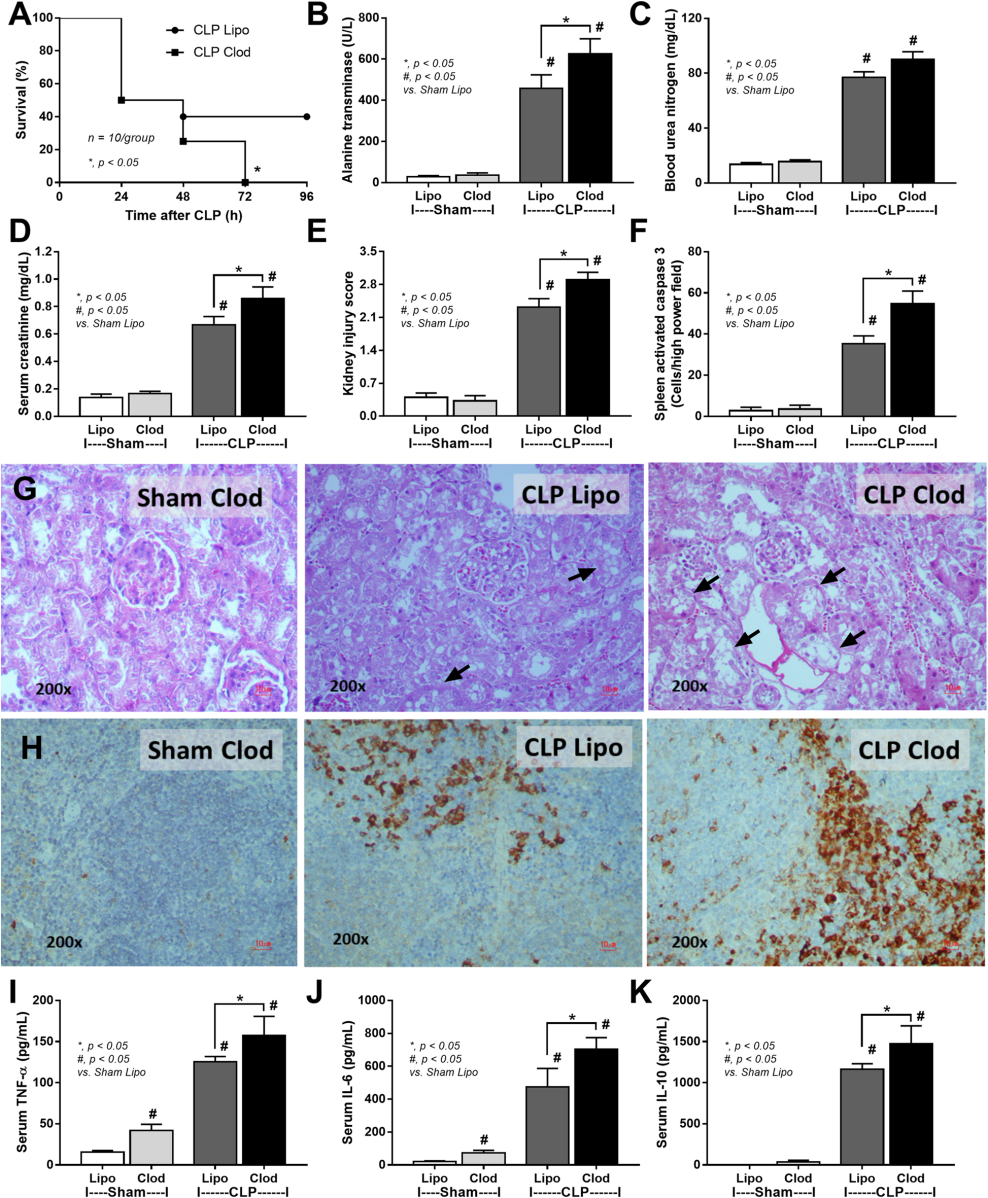
Characteristics of mice with liposomal clodronate (Clod) or liposome control (Lipo) after sham or cecal ligation and puncture (CLP) surgery as indicated by survival analysis (**A**) and several parameters at 24 h post-surgery, including liver injury (serum alanine transaminase) (**B**), kidney damage (blood urea nitrogen, serum creatinine and kidney injury score) (**C**–**E**), spleen apoptosis (activated caspase 3) with the representative pictures of Hematoxylin and Eosin (**H**, **E**) stained renal histology and spleen caspase 3 immunohistochemistry (**F**–**H**) and serum cytokines (TNF-α, IL-6 and IL-10) (**I**–**K**) are demonstrated (n = 10/group for survival analysis and n = 6–8/group for other parameters). Arrows indicate tubular vacuolization in renal histology.

**Figure 5:**
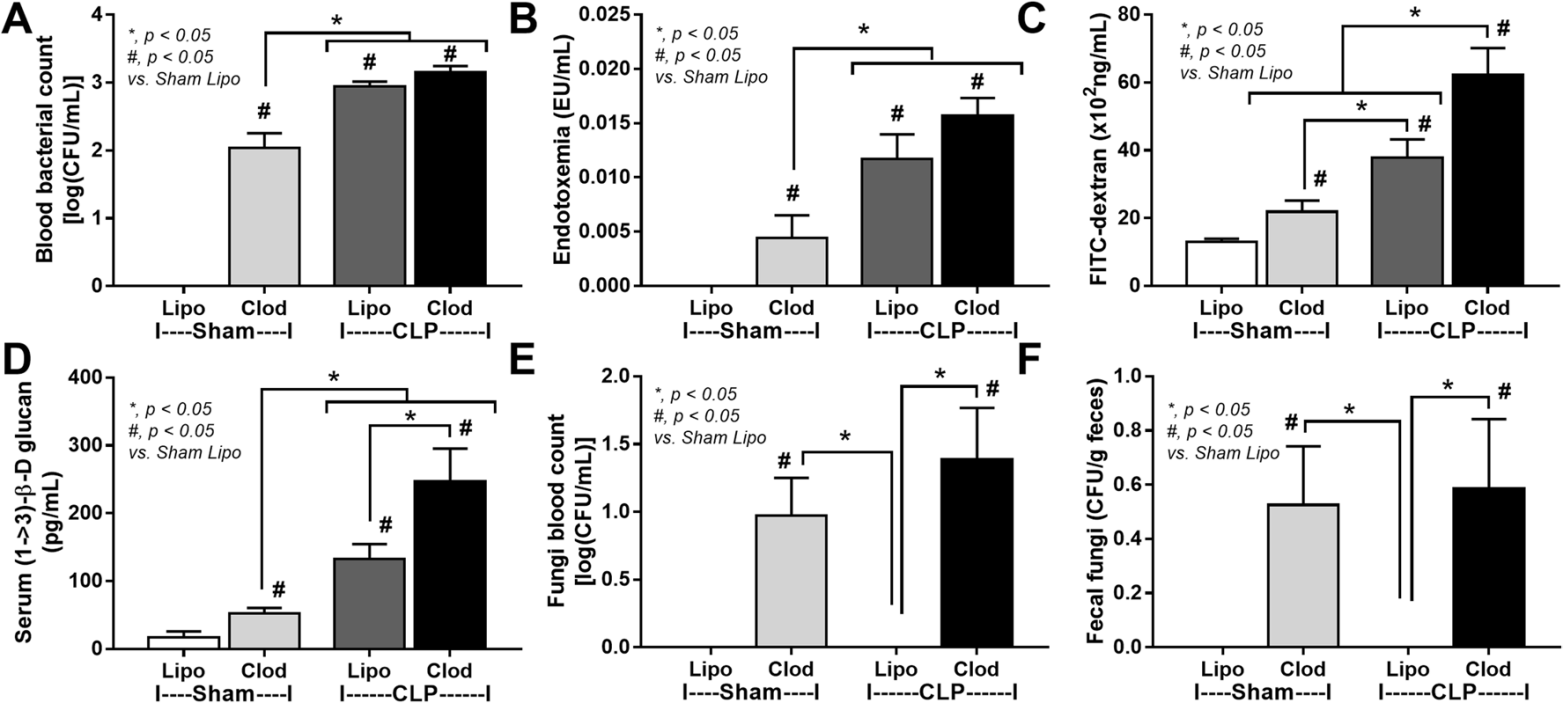
Characteristics of mice with liposomal clodronate (Clod) or liposome control (Lipo) after sham or cecal ligation and puncture (CLP) surgery as evaluated by blood bacterial count (**A**), endotoxemia (**B**), gut permeability defect (FITC-dextran assay, serum (1 → 3)-β-d-glucan and fungal culture from blood) (**C**–**E**) and fungal abundance in feces (**F**) are demonstrated (n = 6–8/group).

With PCR from fecal samples, the abundance of total bacteria, Gram-negative bacteria, pathogenic bacteria (*Klebsiella* spp., *Salmonella* spp., and *Bacteroides *spp.)^34^, but not potentially beneficial bacteria to the host (*Lactobacillus* spp. and *Akkermansia* spp.)^25,29,30^, at 18 h post-CLP was higher than the baseline (Supplement Fig. S1). Likewise, the abundance of total bacteria, Gram-negative bacteria, and most pathogenic bacteria (except for *Klebsiella* spp.), but not the beneficial bacteria, at 18 h post-CLP was higher than 6 h post-CLP (Supplement Fig. S1). Hence, most sepsis parameters were determined at 18 h post-CLP. As such, sepsis was more severe in macrophage-depleted CLP mice than control CLP mice as indicated by survival analysis (Fig. 4A), liver injury (alanine transaminase) (Fig. 4B), renal damage (blood urea nitrogen (Fig. 4C), serum creatinine (Fig. 4D), kidney injury score and histology (H&E staining) (Fig. 4E, G), spleen apoptosis (activated caspase 3) and immunohistochemistry staining (Fig. 4F, H), serum cytokines (TNF-α, IL-6, and IL-10) (Fig. 4I–K) but not bacteremia and endotoxemia (Fig. 5A, B). Additionally, gut permeability defect as demonstrated by FITC dextran assay, serum (1 → 3)-β-d-glucan (BG; the pathogen molecules of fungi) and fungemia (Fig. 5C–E) in macrophage-depleted sepsis mice were higher than liposomal-control sepsis mice, possibly due to fecal fungi that detectable (by culture) only in macrophage-depleted mice (Fig. 5F). Interestingly, macrophage depletion in mice without sepsis (clodronate-administered sham) demonstrated mildly elevated serum cytokines (TNF-α and IL-6) without organ injury (Fig. 4B–K) possibly due to gut permeability defect (gut leakage) as indicated by fungemia (with β-glucanemia) and bacteremia (with endotoxemia), partly from an impact of increased fecal fungi (Fig. 5A–F). These data supported the impact of macrophages on the control of gut fungi and intestinal integrity^16^. Of note, fungal abundance in blood and in feces between clodronate-administered mice after sham and CLP were not different (Fig. 5E–F) suggesting that sepsis did not enhance fecal fungi despite an increase in severity of gut leakage (FITC-dextran) (Fig. 5C).

**Figure 6:**
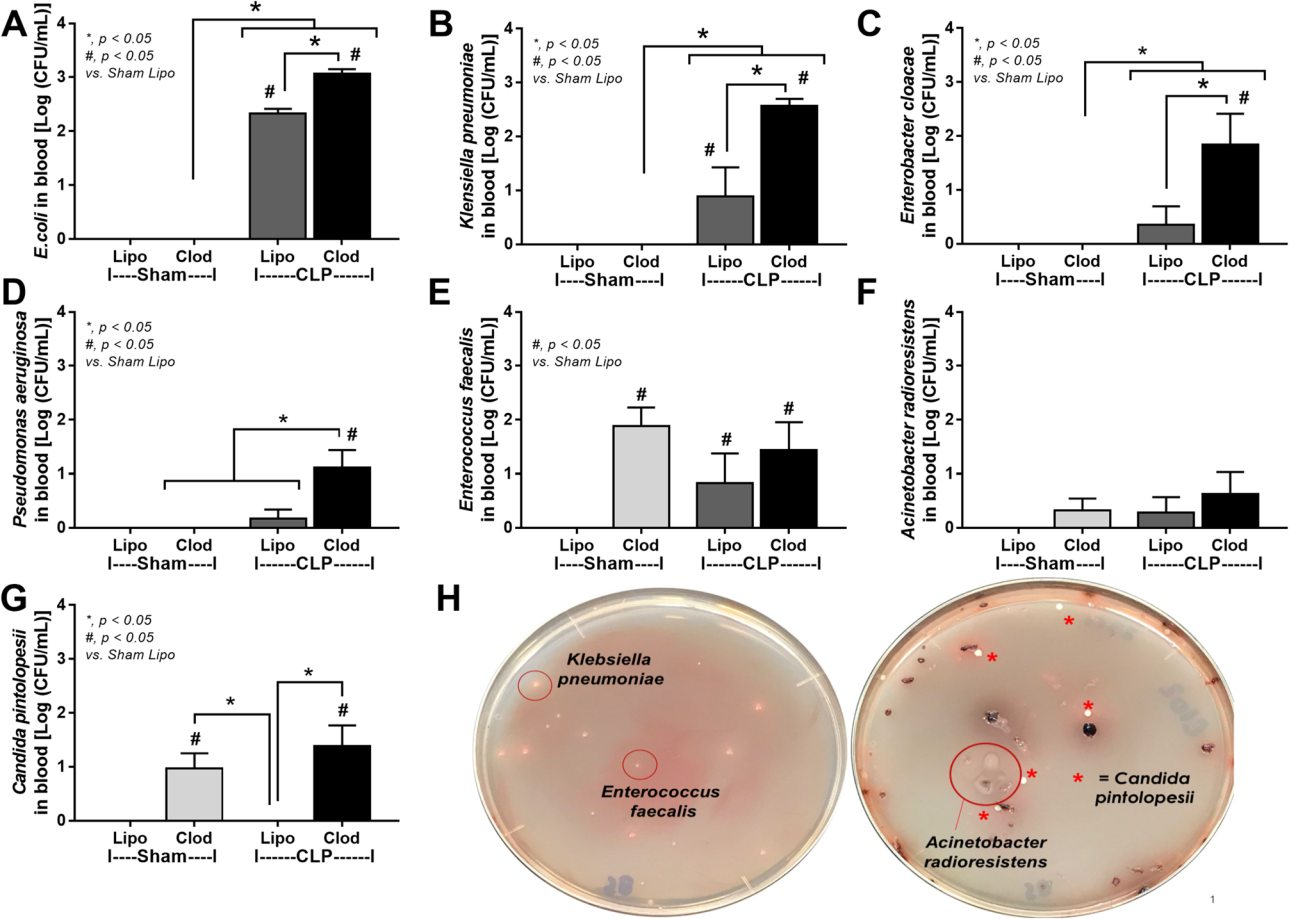
Characteristics of mice with liposomal clodronate (Clod) or liposome control (Lipo) after sham or cecal ligation and puncture (CLP) surgery as evaluated by organism identification (bacteria and fungi) from colony characteristics in blood agar using mass spectrometry analysis (**A**–**G**) are demonstrated (n = 6–8/group). The representative of the blood agar culture plates of CLP mice (**H**) demonstrated the plate with different bacterial colonies without fungal colonies (left side) and the mixed bacterial and fungal colonies (right side). The red circles identify bacterial colonies (using mass spectrometry analysis). The star symbols indicate the colonies of *Candida pintolopesii*.

Additionally, bacterial identification by proteomic analysis based on colony morphologies demonstrated the dissimilarity of organisms from blood culture between sham control (liposome-administered sham) and CLP mice (Fig. 6A–H). As such, macrophage-depleted CLP mice demonstrated the higher abundance in blood of Gram-negative pathogenic bacteria (*E. coli*, *K. pneumoniae*, *Enterobacter cloacae,* and *Pseudomonas aeruginosa*) (Fig. 6A–D) and *C. pintolopesii* (Fig. 6G) without the difference on *E. faecalis* (Gram-positive bacteria) and *Acinetobacter radioresistens* (Fig. 6E, F) when compared with control CLP. Blood organisms were not detectable in liposomal sham control but *E. faecalis* and *C. pintolopesii* were isolated from macrophage-depleted sham mice (Fig. 6A–G) supporting macrophage-depletion-induced gut leakage. However, Candidemia levels between sham and CLP in macrophage-depleted mice were not different (Fig. 6G) implying a less impact of sepsis on the severity of Candidemia.

**Figure 7:**
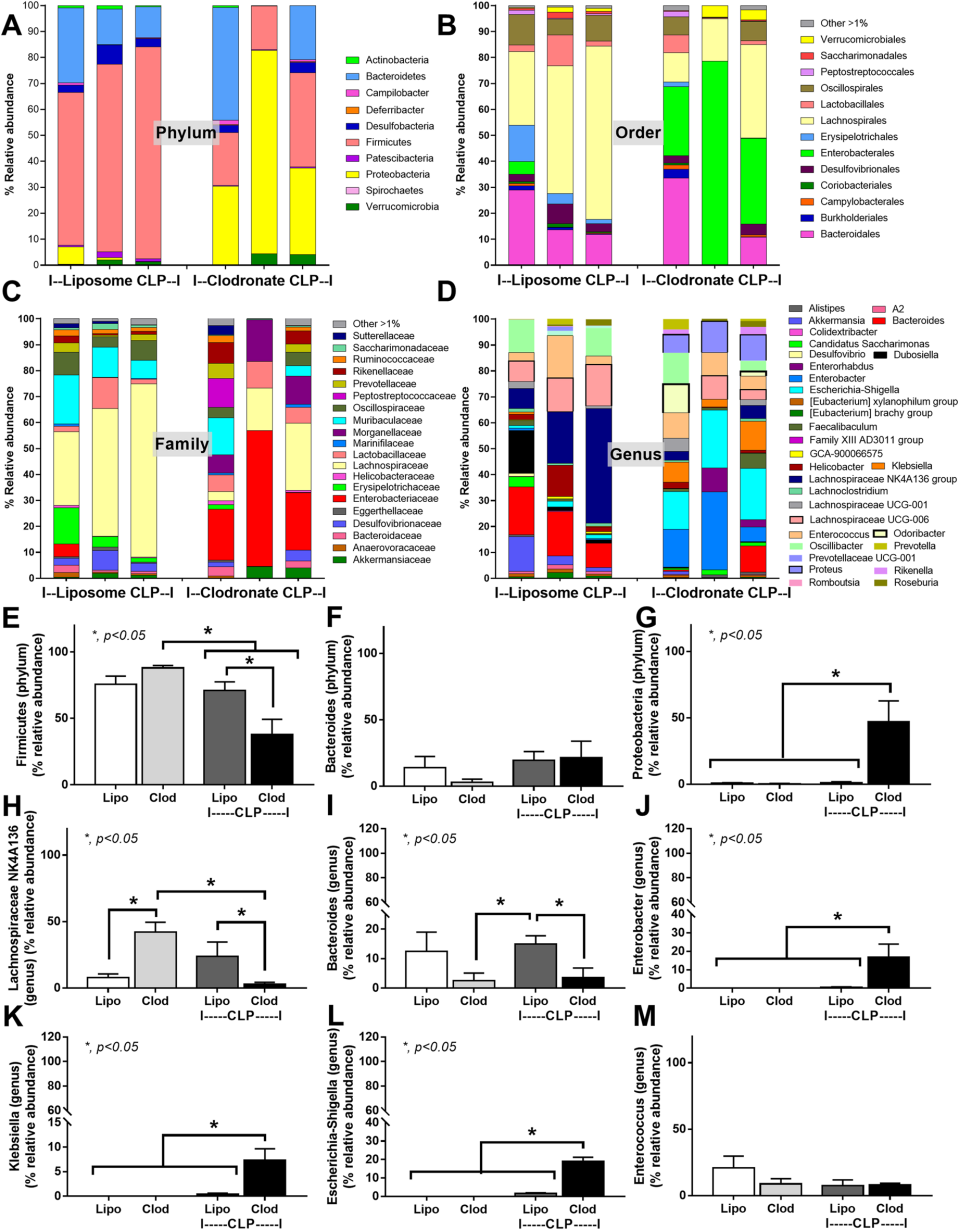
Bacterial microbiome analysis from feces of mice with liposomal clodronate (Clod) or liposome control (Lipo) at 24 h after cecal ligation and puncture (CLP) surgery as indicated by the relative abundance from individual samples in phylum, order, family and genus (**A**–**D**) with graph presentation of the abundance (**E**–**M**) are demonstrated.

**Figure 8:**
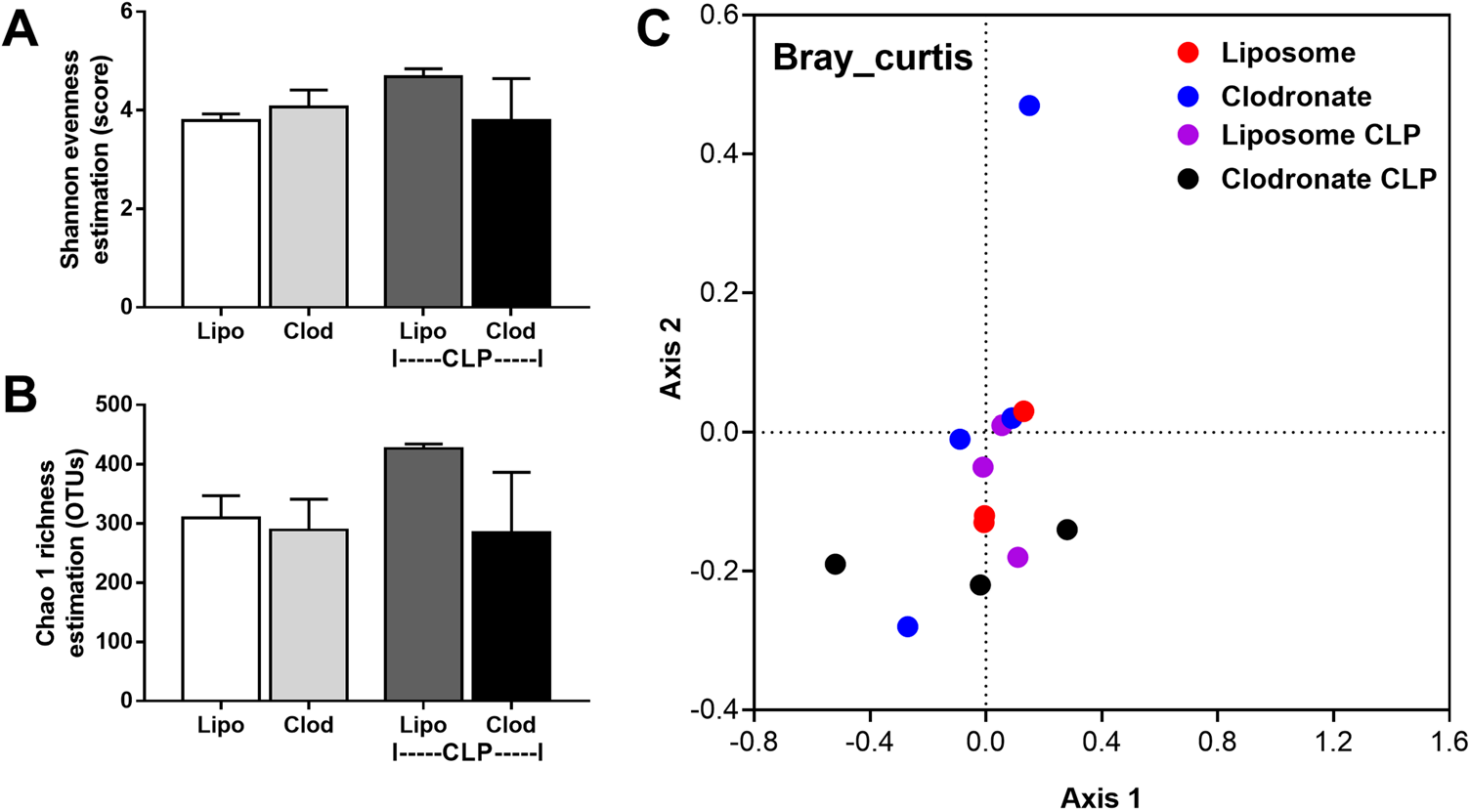
Bacterial microbiome analysis from feces of mice with liposomal clodronate (Clod) or liposome control (Lipo) at 24 h after cecal ligation and puncture (CLP) surgery as indicated by alpha diversity (Shannon and Chao) (**A**, **B**) and beta diversity (Bray Curtis) (**C**) are demonstrated.

The multiple bacterial species with only a single fungal species from blood culture (Fig. 6A–H) in macrophage-depleted sepsis mice and a less variation of fecal fungi (mycobiota analysis) macrophage-depleted control mice (Fig. 2A–G) implied the more profound alteration in gut bacteria than the fungi. Then, bacterial microbiome analysis, but not mycobiota analysis, was further evaluated. In macrophage-depleted CLP mice, fecal bacteria in phylum Firmicutes (the dominant fecal bacteria in healthy mice^25,29,30^) and Proteobacteria (pathogenic bacteria^25,29,30^) were decreased and increased, respectively, while the abundance of Bacteroides phylum was not different (Fig. 7A–G) when compared with control CLP mice. In genus-level analysis, macrophage-depleted CLP mice demonstrated the lesser of several Lachnospiraceae (Firmicutes bacteria) and *Bacteroides* spp. (possibly *Bacteroides fragilis* in Bacteroides bacteria) with the higher *Enterobacter* spp., *Klebsiella* spp., and *Escherichia-Shigella* spp. (Proteobacteria) when compared with the control CLP (Fig. 7A–L). Meanwhile, the abundance of *Enterococcus* spp. (possibly *E. faecalis*) in macrophage-depleted CLP mice was not different from control CLP (Fig. 7M) in similar to the blood culture of both groups (Fig. 6E). Likewise, alpha and beta diversity in CLP mice with and without macrophage depletion was also non-different (Fig. 8A–C). Notably, Proteobacteria were not detectable in the microbiome analysis of mice without surgery, supporting an overgrowth of gut pathogenic bacteria in sepsis^38^. Also, Firmicutes bacteria (especially Lachnospiraceae group) in macrophage-depleted mice without sepsis was higher than macrophage-depleted sepsis mice (Fig. 7E, H). Meanwhile, Proteobacteria (Enterobacter, Klebsiella, Escherichia-Shigella) was lower than macrophage-depleted sepsis mice (Fig. 7G, J–L), implying a more severe gut dysbiosis after macrophage depletion.

### An impact of gut fungi on a selected growth of some bacteria and the stimulation on neutrophils and enterocytes, an influence of gut fungi on sepsis severity

**Figure 9:**
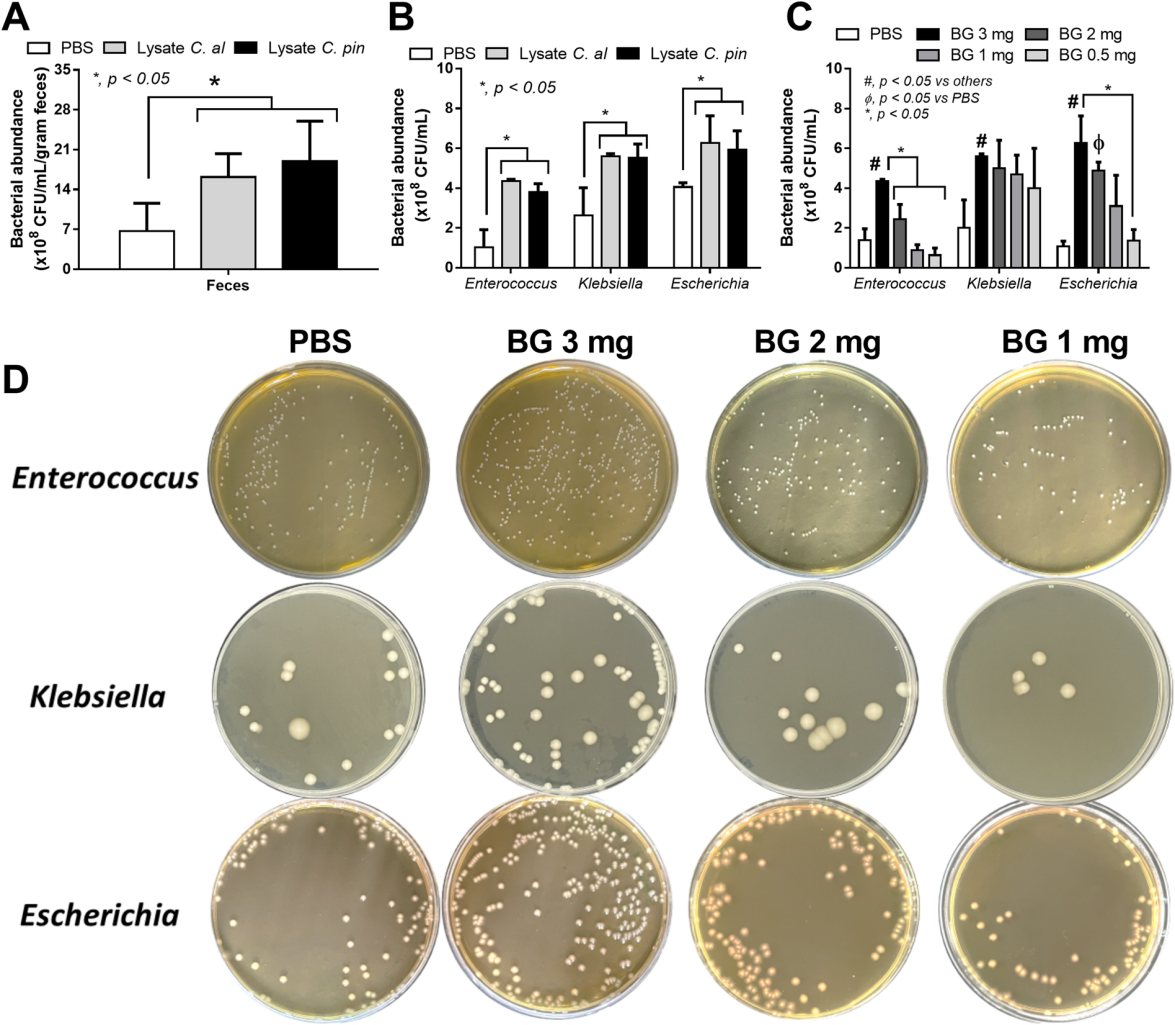
Bacterial abundance after incubation with phosphate buffer solution (PBS) or the lysate of *Candida albicans* (*C. alb*) or *Candida pintolopesii* (*C. pin*) in the samples using normal mouse feces (**A**) or isolated bacteria from blood of clodronate-administered CLP mice, including *Enterococcus faecalis*, *Klebsiella pneumoniae* and *Escherichia coli*, (**B**) are demonstrated (n = 8/group). Also, bacterial abundance of these isolated bacteria after incubated with PBS or (1 → 3)-β-d-glucan (BG) with the different concentrations per blood agar culture plate (**C**) (n = 7/group) and the representative pictures of the blood agar culture plates (**D**) are demonstrated.

**Figure 10:**
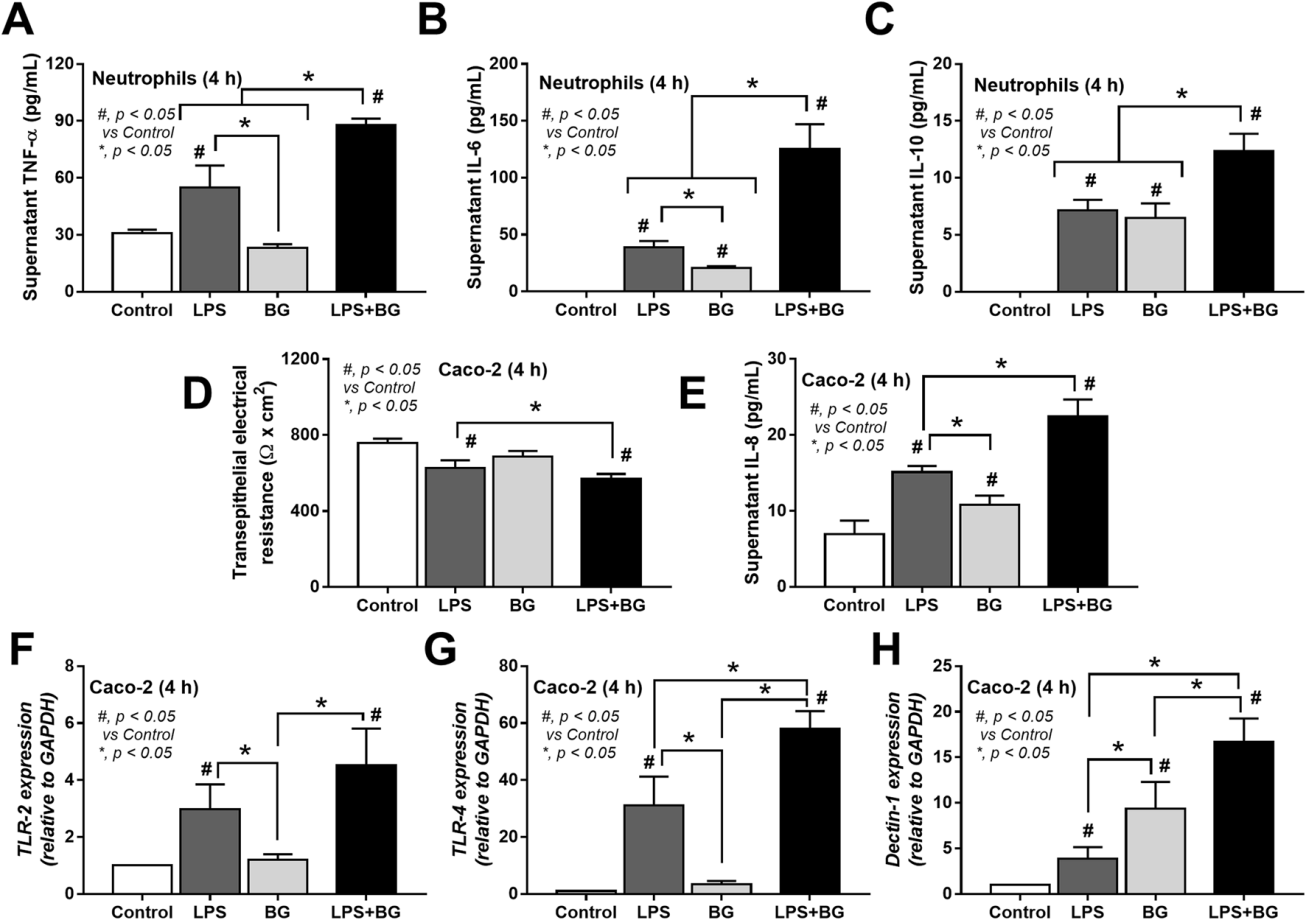
Characteristics of cells after incubation with media control (control), lipopolysaccharide (LPS), (1 → 3)-β-d-glucan (BG) or LPS with BG (LPS + BG) as indicated by supernatant cytokines (TNF-α, IL-6 and IL-10) from human neutrophils (**A**–**C**), the injury of intestinal cells (Caco-2 cells) using transepithelial electrical resistance (TEER) and supernatant IL-8 (**D**, **E**) are demonstrated (independent triplicated experiments were performed), and stimulated Caco-2 cells was determined genes expression including TLR-2, TLR-4, and Dectin-1 (**F**–**H**).

*Candida* in the gut might be a source for bacterial fermentation as BG, a major source of the fungal cell wall, could enhance the growth of some bacteria^6^ and glucan digestible bacteria are identified^39^. Here, the ex vivo incubation of feces from control mice (non-sepsis) with the lysate of heat-killed fungi using *C. albicans* (ATCC 90028) or *C. pintolopesii* (isolated from the blood of Clodronate-administered mice) was performed. Interestingly, the lysate of both fungi increased bacterial abundance in fecal samples when compared with the incubation by PBS control (Fig. 9A). Due to the positive blood culture of several organisms from Clodronate-administered CLP mice, these bacteria (*E. faecalis*, *K. pneumoniae*, and *E. coli*) were incubated with the fungal lysate and the lysate facilitated the growth of these bacteria (Fig. 9B). Likewise, the addition of BG, especially in a high concentration, also augmented these bacteria (Fig. 9C, D). However, gut fungi might  not only influence bacteria but also enterocytes and other immune cells. Then, human neutrophils and Caco-2 enterocyte cell line were incubated with LPS with and without purified BG. In neutrophils, BG alone mildly induced supernatant IL-6 and IL-10, while LPS elevated all cytokines (TNF-α, IL-6, and IL-10) when compared with the control (Fig. 10A–C). Nevertheless, an incubation of LPS with BG induced higher levels of all neutrophil cytokines than LPS activation alone (Fig. 10A–C). In enterocytes, the stimulation by LPS alone or BG alone mildly elevated supernatant IL-8 without an effect on enterocyte damage (TEER), while LPS with BG induced the higher supernatant IL-8 with a decrease in enterocyte integrity (TEER) (Fig. 10D, E). Additionally, LPS upregulated the gene expression of *TLR-2, TLR-4*, and *Dectin-1* in enterocytes, while BG enhanced only *Dectin-1* (Fig. 10F–H). With LPS plus BG (LPS + BG), *TLR-4* and *Dectin-1*, but not *TLR-2* expression, was more prominent than LPS alone (Fig. 10F–H) implying that the LPS + BG synergy partly process through the co-stimulation of *TLR-4* and *Dectin-1* on enterocytes^40,41^. These data also suggested the importance of fungal molecules in enhancing both local intestinal (enterocytes) and systemic inflammation (neutrophils) in macrophage depleted mice.

## Discussion

The enhanced severity of sepsis in macrophage-depleted CLP mice through the increased abundance of gut fungi illustrated the importance of macrophages and gut fungi in sepsis.

### Increased fecal fungi with impaired gut permeability in macrophage-depleted mice facilitated sepsis severity, a 2-hit sepsis model due to fungemia and bacteremia

**Figure 11:**
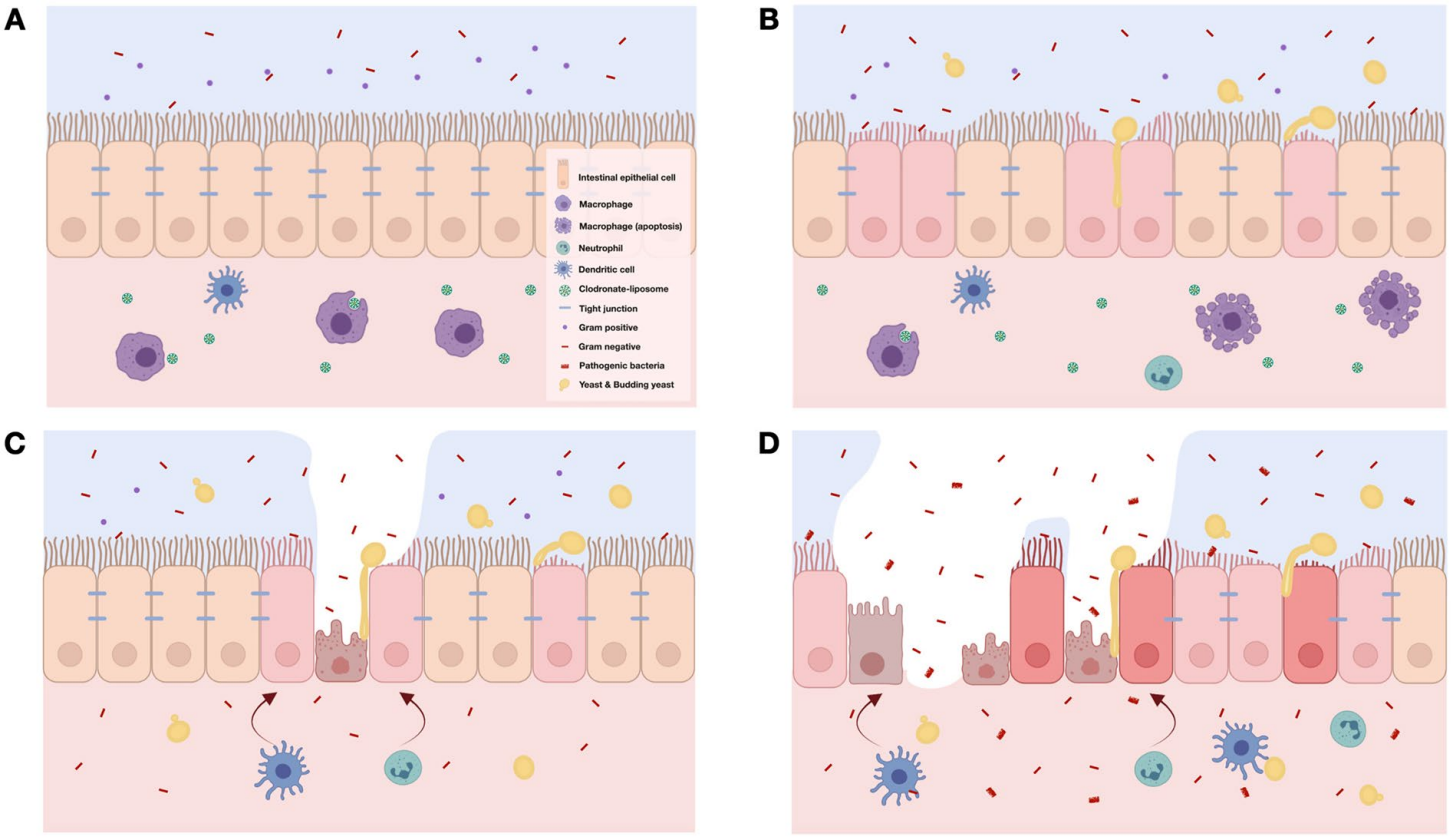
The working hypothesis demonstrates a balance between immune responses, especially macrophage function, versus intestinal microbes in the mice with intact macrophage (**A**). Clodronate induces macrophage apoptosis and causes an overgrowth of gut fungi (*Candida pintolopesii*) in a later stage (**B**). In Clodronate-administered sham mice, macrophage depletion causes fungal overgrowth and enhances bacteria with a less virulence, particularly *Enterococcus faecalis*, in gut causing a mild degree of gut permeability defect through enterocyte tight junction damage or enterocyte cell death (**C**). With cecal ligation and puncture (CLP) sepsis, sepsis induces an overgrowth of pathogenic bacteria (sepsis-induced gut dysbiosis), especially Gram-negative bacteria (Proteobacteria), and gut fungi which facilitates growth of some other strains of pathogenic bacteria results in a more severe gut leakage, bacteremia of the high virulence microbes and severe sepsis (**D**). The picture is created by BioRender.com (https://app.biorender.com/illustrations).

Liposomal chlodronate effectively depleted peripheral blood monocytes and intestinal macrophages supported the previous publications^42,43^. The lessor fungal abundance of mouse feces than human stool^6–8,44,45^ and the easier fungal detection in patient feces, especially with antibiotics administration^46,47^, are previously mentioned. Fecal fungi in mice without macrophage depletion (non-sepsis and sepsis) was negative by culture and become positive after macrophage depletion, supporting the role of macrophage in control of gut fungi. Indeed, macrophage depletion (non-sepsis) possibly more prominently affects fungi than bacteria in the gut, as it induced the profound Ascomycota fungi (a group of pathogenic fungi), but not increased pathogenic bacteria (Proteobacteria). However, enhanced fecal Ascomycota with reduced Firmicutes (a group of beneficial bacteria on gut integrity^25,29,30^) in non-sepsis macrophage-depleted mice induced gut dysbiosis that severe enough for causing gut permeability defect (gut leakage)^48^, partly be due to enterocyte damage by fungi (for example, germ tube formation from *Candida*)^49^. The fungemia and bacteremia in non-sepsis macrophage-depleted mice might be the first hit injury that worsens sepsis, similar to other 2-hit sepsis models^50^, as macrophage-depleted CLP mice were more severe than control CLP (Fig. 11A–D). Despite the well-known gut fungal growth-facilitation from chronic intestinal inflammation due to the diminished competition by commensal bacteria^6,51^, CLP did not enhance fecal fungal burdens (perhaps because of too short duration). Although fecal burdens of fungi (by culture and observed OTUs) and bacteria (observed OTUs) between macrophage-depleted mice with sham versus CLP were not different, gut leakage in CLP mice was more severe than sham mice, perhaps due to the intestinal hypoxia and gut pathogens after sepsis^30,52^.

### Fecal fungi enhanced the growth of some high virulence bacterial strains in the gut that facilitated bacteremia and worsened sepsis in macrophage-depleted mice

Facilitated growth of some bacteria in gut dysbiosis^53^ , such as the promote growth of *P. aeruginosa* with the presence of *Candida* spp. in gut^6^, and the importance of macrophages in the control of pathogenic bacteria^54^ are previously mentioned. An ability on the digestion of BG, a major molecule of the fungal cell wall, in some gut bacteria might be responsible for the selected growth of the specific gut bacteria^6^, while prokaryotic polyphosphates in some bacteria facilitate the severity of infection through the reduced anti-microbial activities of macrophages^54^. With macrophage depletion, bacteremia in sham and CLP mice were mainly *E. faecalis*, the commensal bacteria^55^, and Gram-negative pathogenic bacteria, respectively. While *E. faecalis* in sham mice mildly increased serum cytokines without organ injury, Gram-negative bacteria in CLP caused mortality and organ damage supporting the difference in bacterial virulence^56^. Although commensal organisms, including *E. faecalis*, are necessary for host immune maturation and gut-barrier maintenance^57^, they can also activate inflammation during gut leakage^31^. Despite a similarity between the strains of isolated Gram-negative bacteria between CLP mice with or without macrophage depletion, the abundance of pathogenic bacteria in the blood (blood culture) and in feces (microbiome analysis) was more profound in macrophage-depleted mice. On the other hand, prominent fecal fungi in macrophage-depleted mice (sham and CLP) were Ascomycota (a group of *Candida* spp.) in accordance with the isolated *C. pintolopesii* from mouse blood. These data suggest that macrophage depletion facilitated *Candida* growth that enhanced growth of *E. faecalis* and Proteobacteria in sham and CLP mice, respectively. Perhaps, gut *Candida* in sham facilitates commensal bacteria (*E. faecalis*), while gut inflammation in CLP induces growth of pathogenic bacteria^58,59^ that are augmented by gut *Candida*. Indeed, sepsis, even non-gastrointestinal infection, enhances gut-pathogenic bacteria and leaky gut^60,61^, partly through the stress-induced gut dysbiosis^62,63^. Because some bacteria from macrophage-depleted mice might be augmented by fungi (fungal digesters)^39^, isolated bacteria from the blood of macrophage-depleted CLP mice were further tested. Accordingly, the abundance of the isolated *E. faecalis*, *K. pneumoniae* and *E. coli* in culture was enhanced by fungal lysate or (1 → 3)-β-d-glucan (BG; a major molecule of the fungal cell wall), suggesting assistance of gut fungi on bacterial growth possibly due to the fungal digestion abilities^64^. As such, enhanced growth of *Enterococcus* spp. in culture media supplemented with β-glucans^65,66^ and β-1,3-glucanase producing Proteobacteria are reported^39^. To understand more about the mechanisms of these relationships, further studies are warranted.

### Increased gut fungi in macrophage-depleted mice, an impact on enterocyte and neutrophils, the local and systemic inflammation, respectively

The enhanced level of BG in gut content, through increased fecal fungi by oral administration, worsens sepsis severity through elevated systemic inflammation from gut translocation of BG is previously described^26,45,67,68^. Here, macrophage depletion induced the spontaneous elevation of fecal fungi in mice, in particular *C. pintolopesii* (the previously known *Candida* non-albicans in mouse guts^69^) that selectively enhanced some strains of bacteria (gut dysbiosis) and causing gut translocation of LPS and BG. Likewise, LPS together with BG (LPS + BG) additively increased enterocyte- and neutrophil-mediated inflammation compared with LPS or BG alone which worsened sepsis-hyper inflammation. The presence of BG together with LPS in the gut might enhance the pro-inflammatory responses of enterocytes compared with the activation by each molecule alone possibly through the co-stimulation of TLR-4 and Dectin-1 in similar to other cells^26,45,67^. Indeed, intravenous injection of BG together with LPS induces a higher level of serum cytokines when compared with the administration by each molecule alone^40^ supporting the synergy of BG upon LPS responses in several mouse models^7,45,70^.

Subsequently, the prominent enterocyte inflammation might induce gut barrier defects and increase LPS and BG in portal vein^5^ that stimulate several cells in the liver, including hepatic sinusoidal endothelium and Kupffer cells (the first cell populations that come into contact with portal vein-derived molecules from the gut)^71^ leading to the increased serum cytokines (but not enough to elevate liver enzyme). More studies on the liver responses after gut barrier defect are interesting. Nevertheless, an extreme macrophage inhibition from the emerging macrophage interference in several diseases (cancers, autoimmune diseases, and sepsis^19,72^ might enhance fecal fungi and worsen sepsis severity. Some biomarkers (serum BG or fecal fungi) during macrophage inhibition might be useful. Further studies are interesting.

In conclusion, macrophage depletion enhanced fecal *Candida* overgrowth, fecal pathogenic bacteria, intestinal barrier damage, and gut translocation of LPS and BG that additively worsened sepsis severity. Fecal fungal burdens and gut leakage parameters are possible beneficial biomarkers during the treatment with macrophage inhibition. More studies on these topics are interesting.

## Supplementary Information

Supplementary Legends.

Supplementary Figure S1.

## Data Availability

The datasets presented in this study can be found in online repositories. The names of the repository/repositories and accession number(s) can be found below: NCBI (accession: PRJNA765503).
