# Supplementary material for: Macrophage depletion alters bacterial gut microbiota partly through fungal overgrowth in feces that worsens cecal ligation and puncture sepsis mice
Source: Sci Rep. 2022 Jun 4;12:9345. doi: 10.1038/s41598-022-13098-0 (PMC9167291; doi:10.1038/s41598-022-13098-0)
Supplement: Supplementary file 1 — Supplementary Legends. [file 41598_2022_13098_MOESM1_ESM.docx]

**Supplement figure 1.** The abundance of total bacteria and some pathogenic bacteria from mouse feces after cecal ligation and puncture (CLP) surgery, including total bacteria, total Gram-negative bacteria, *Klebsiella* spp., *Salmonella* spp., Bacteroides spp., Lactobacillus spp., and *Akkermansia* spp., as measured by real-time polymerase chain reaction (PCR) as represented by the difference between the detected cycle threshold (Ct) (measured Ct) with the Ct of negative value (Ct value at 40) (A-G) are demonstrated (n = 4/ time-point).
