# Supplementary figures and images for: Macrophage depletion alters bacterial gut microbiota partly through fungal overgrowth in feces that worsens cecal ligation and puncture sepsis mice
Source: Sci Rep. 2022 Jun 4;12:9345. doi: 10.1038/s41598-022-13098-0 (PMC9167291; doi:10.1038/s41598-022-13098-0)

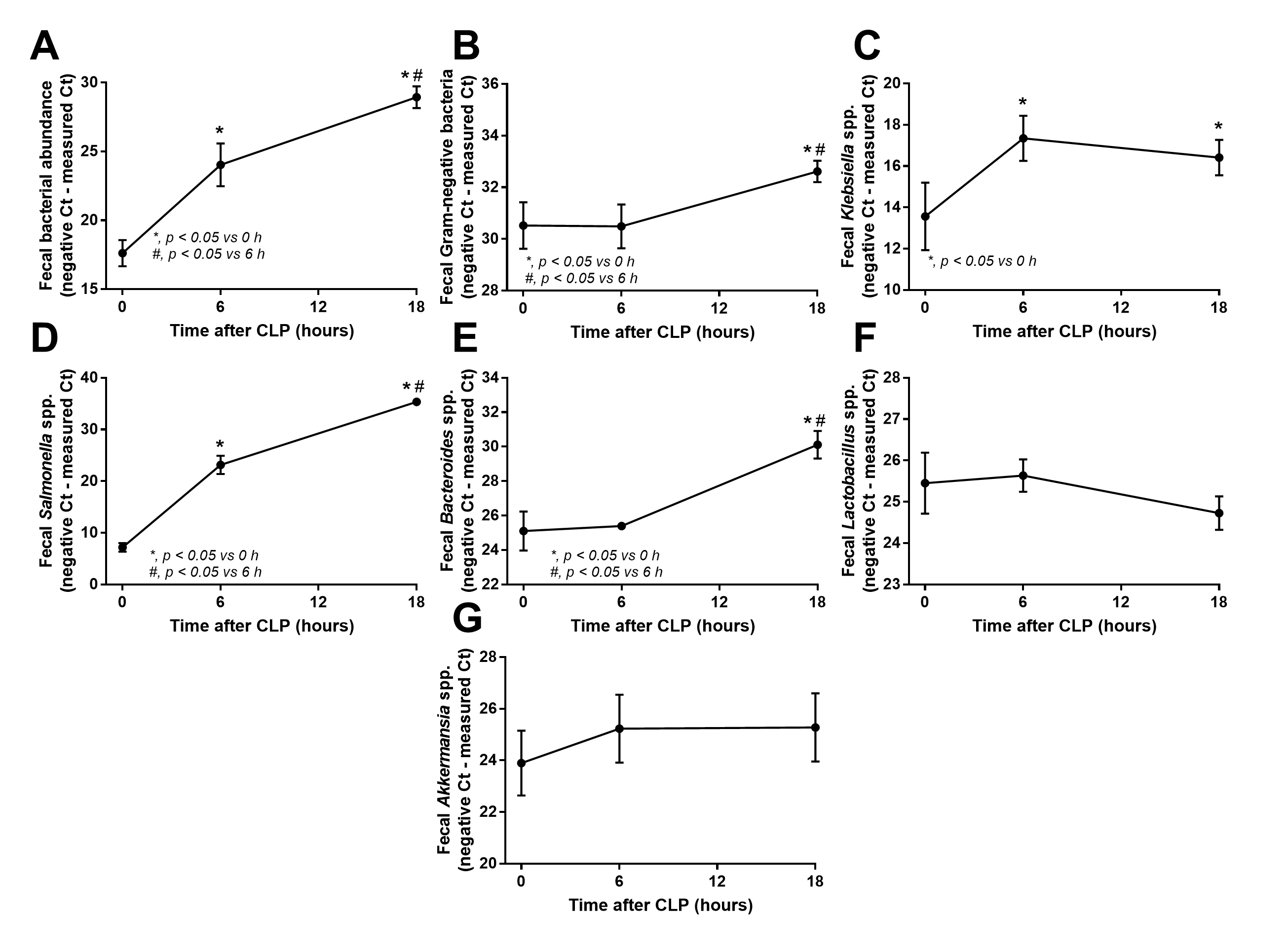

Supplement: Supplementary file 2 — Supplementary Figure S1. [file 41598_2022_13098_MOESM2_ESM.tif]
